# Character Decomposition and Transposition Processes of Chinese Compound Words in Rapid Serial Visual Presentation

**DOI:** 10.3389/fpsyg.2017.00483

**Published:** 2017-03-31

**Authors:** Hong-Wen Cao, Ke-Yu Yang, Hong-Mei Yan

**Affiliations:** ^1^Key Laboratory for NeuroInformation of Ministry of Education, Center for Information in Medicine, University of Electronic Science and Technology of ChinaChengdu, China; ^2^Department of Military Psychology, School of Psychology, Third Military Medical UniversityChongqing, China

**Keywords:** Chinese compound words, character order, character decomposition, character transposition, transposition cost

## Abstract

Character order information is encoded at the initial stage of Chinese word processing, however, its time course remains underspecified. In this study, we assess the exact time course of the character decomposition and transposition processes of two-character Chinese compound words (canonical, transposed, or reversible words) compared with pseudowords using dual-target rapid serial visual presentation (RSVP) of stimuli appearing at 30 ms per character with no inter-stimulus interval. The results indicate that Chinese readers can identify words with character transpositions in rapid succession; however, a transposition cost is involved in identifying transposed words compared to canonical words. In RSVP reading, character order of words is more likely to be reversed during the period from 30 to 180 ms for canonical and reversible words, but the period from 30 to 240 ms for transposed words. Taken together, the findings demonstrate that the holistic representation of the base word is activated, however, the order of the two constituent characters is not strictly processed during the very early stage of visual word processing.

## Introduction

Visual word identification is a basic process in reading that requires readers to assess the identity and position of the letters in a word (Inhoff, 1990; Besner and Humphreys, 1991; Davis, 2010). More interestingly, skilled readers can easily understand text with letter transpositions in an alphabetic writing system (Davis, 2003). However, Rayner et al. (2006) claimed that text with letter transpositions always carry a cost during reading. Using the boundary paradigm, Angele and Rayner (2013) examined morpheme order transposition in reading English compound words, they found that the readers could obtain preview benefit from both normal and reversed words. Additionally, researchers used unprimed and masked primed lexical decisions to examine letter transposition and argued that words can be accessed both through a direct whole-word route and via a morphological decomposition route (Beyersmann et al., 2012).

Analogous to English, character order information is also important during Chinese reading. Bai et al. (2011) explored the time course of compound word processing in Chinese during a lexical decision task. Reversible words, non-reversible words and pseudowords were employed in that study. The findings indicated that both character combinations as well as access to the individual constituent meanings interfered with the processing of reversible and non-reversible words. They argued that structural reversibility in Chinese word formation has an impact on target identification. Several researchers have explored character order encoding in isolated word processing or Chinese sentence reading using a masked priming paradigm and a gaze-contingent display-change paradigm. They found that character position encoding was not strict but occurred at an early processing stage in Chinese reading (Gu et al., 2015). However, it should be noted that previous studies only claimed that the character order information is encoded at the initial stage of Chinese word processing, the exact time course of character decomposition and transposition processes of Chinese compound words remains underspecified.

In normal reading, readers fixate at a certain word, and then make a saccade from the current fixation position to the next point of fixation, but the processing of the character and lexical information is a temporal course. Rapid serial visual presentation (RSVP) is a well-established method for studying the time course of language processing and reading (Potter, 1984). Reading in RSVP is fairly equivalent to conventional reading when presentation is at an adequate rate, such as 12 words per second (Petrick and Pottes, 1979; Juola et al., 1982). For skilled readers, the phonological and orthographic information could be activated automatically at 30- and 60-ms presentation durations, due to the precision and redundancy of their lexical representations (Booth et al., 1999). In the dual-target RSVP tasks, when two completely unrelated Chinese characters are presented in an RSVP sequence with 60 ms per item, the identification of the second target character is severely impaired if it occurs within approximately 240 ms after the first target character. This phenomenon is known as the attentional blink (AB; Raymond et al., 1992). The ABs are eliminated when two characters could be integrated into a single compound word regardless of their orders (Cao H. et al., 2016). Owing to the temporal characteristics of the RSVP paradigm, participants may reverse the temporal order of the two targets, namely T1 is reported as T2, and T2 is reported as T1 (Chun and Potter, 1995; Spalek et al., 2006). The proportion of order reversals for the two targets showed a substantial decrement from Lag 1 (no intervening item) to Lag 3 (two intervening items) during the AB (Bowman and Wyble, 2007; Wyble et al., 2009). Moreover, our previous studies on the character transpositions in the left and right visual fields also revealed that the order of the foveally presented Chinese words was more likely to be reversed at the duration of 100 ms (Cao H.W. et al., 2016). As indicated above, it is still unclear about the time course of character decomposition and transposition processes of Chinese words when the dual targets appear very fast, for example, at 30 ms per character with no inter-stimulus interval.

Taken together, by manipulating the stimulus onset asynchrony (SOA, 30–240 ms) and the morpheme position within two-character compound words (canonical, transposed, and reversible words) and pseudowords, the present study set out to further explore two questions. (1) The first is the impact of the character decomposition and transposition processes of Chinese compound words on visual word identification during RSVP reading. If there has been an effect, the accuracy rates of transposed words will be significantly lower than those of canonical and reversible words across all the SOAs. (2) The second is the time course of character order errors during two-character compound words processing. We predict that character order errors occurs mainly within 240 ms.

## Materials and Methods

### Participants

Forty-five native Chinese speakers (20 males and 25 females, their ages ranged from 21 to 34 years, *Mean* = 25.8, *SD* = 3.17) were included in this experimental procedure after giving written informed consent, in agreement with the prior approval (approval number: 00085) of the Ethics and Human Participants in Research Committee at the University of Electronic Sciences and Technology of China in Chengdu, China. All subjects had normal or corrected-to-normal vision and were naive to the purpose of the experiments.

### Apparatus

The experimental program was compiled by MATLAB (MathWorks, Natick, MA, USA) using Psychtoolbox (Brainard, 1997; Pelli, 1997). The stimuli were presented on the center of a display computer with a high-resolution color monitor (1024 × 1280 pixels, 3 × 8 bit RGB, 100 Hz).

### Stimuli

Four types of paired two-character Chinese compound words were used as target stimuli: (1) canonical words, e.g., “
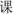
” (class, T1) and “
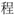
” (procedure, T2), the two targets form a two-character Chinese compound word, which means ‘course’ in order (T1+T2), while meaningless in the reverse order (T2+T1); (2) transposed words, e.g., “
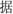
” (evidence, T1) and “
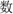
” (number, T2), which is obtained by transposing the position of the constituent characters of the canonical word “
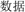
” (T2+T1, means “data”), while it is meaningless in the normal order (T1+T2); (3) reversible words, in which the two characters can constitute two different meaningful words by switching the position of the constituent morphemes, e.g., “
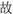
” (old, T1) and “
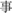
” (thing, T2), which means “story” (T1+T2) in the forward direction and “accident” (T2+T1) in the backward direction; and (4) pseudowords, e.g., “
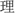
” (reason, T1) and “
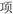
” (item, T2), the two characters form a meaningless pseudoword in both forward and backward conditions. Each condition included 128 stimulus pairs resulting in a total of 512 pairs of Chinese two-character compound words.

All chosen two-character compound words were the most commonly used and had a mean frequency of occurrence of 76.21 (*SD* = 14.48) per million for canonical words, 79.32 (*SD* = 12.77) for reversible words and 71.83 (*SD* = 17.47) for their corresponding canonical words for transposed words according to the Language Teaching, and Research Institute of Beijing Language Institute (1986). One-way analysis of variance (ANOVA) revealed no significant differences for the frequencies of T1 and T2 across all conditions (all *p* > 0.05). The visual complexity (in terms of the number of strokes per character) was matched across each stimulus type. The mean number of strokes for T1 and T2 are 9.71 (*SD* = 2.58) and 9.51 (*SD* = 2.48) for canonical words, 8.41 (*SD* = 2.8) and 8.55 (*SD* = 3.01) for reversible words, 9.32 (*SD* = 2.21) and 9.48 (*SD* = 2.69) for transposed words, 8.01 (*SD* = 2.37) and 8.03 (*SD* = 2.28) for pseudowords, respectively. There were no significant differences in strokes between the two targets among the four conditions (all *p* > 0.05). The distractors consisted of the 100 most frequently used Chinese characters (2–9 strokes), which were irrelevant to the targets in terms of their semantic information.

### Procedure

**FIGURE 1:**
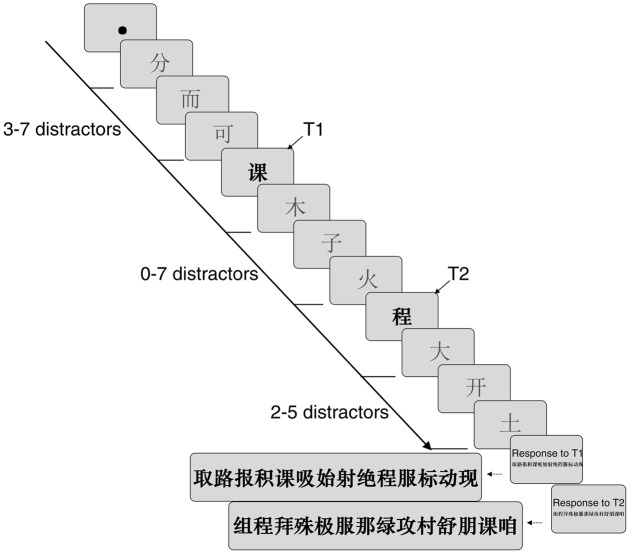
**Sample trial sequences during the experimental paradigm.** The presentation rate was 30 ms/item. The characters chosen as T1 and T2 for the discrimination task were presented in bold, whereas the distractors were displayed in a normal font.

Subjects were tested with a viewing distance of approximately 60 cm, and their head movements were immobilized by forehead and chin rests during the experiment. They were required to maintain fixation on the center of the screen throughout the experiment and were asked to identify the two bold black target characters in the order. During each trial, a fixation dot (0.3° in diameter) appeared for 800 ms in the center of screen. Then, two bold, black font Chinese characters (0.86° × 0.95°, referred to as targets, marked T1 and T2, respectively) were sequentially presented among normal font characters (distractors) in a dual-target RSVP. The presentation rate was 30 ms/item. There were 3–7 distractors that were randomly presented prior to T1. The number of distractors between T1 and T2 systematically varied from 0 to 7, specifically from 30 to 240 ms. Finally, at least 2–5 distractors followed T2. After the stream, the first panel containing 14 bold black Chinese characters was displayed on the screen, and the subjects were instructed to identify T1 in the order in which they saw it by clicking the mouse on it. Note that the 12 Chinese characters were chosen from a set of distractors, some of which could also be integrated into a meaningful word with either T1 or T2. Once T1 was chosen, a second panel with another 14 characters was automatically presented to identify T2. Participants were asked to click the blank area on the panel when they did not see the target characters (**Figure 1**).

The procedure was self-paced. The items on a given trial were randomly generated and were presented only once during the experiment. Each subject performed eight blocks (a total of 512 trials, with 64 trials at each lag). The block order was counterbalanced for each participant and randomized across subjects. All subjects received an initial training of 40 trials before the experimental phase began.

## Results

**FIGURE 2:**
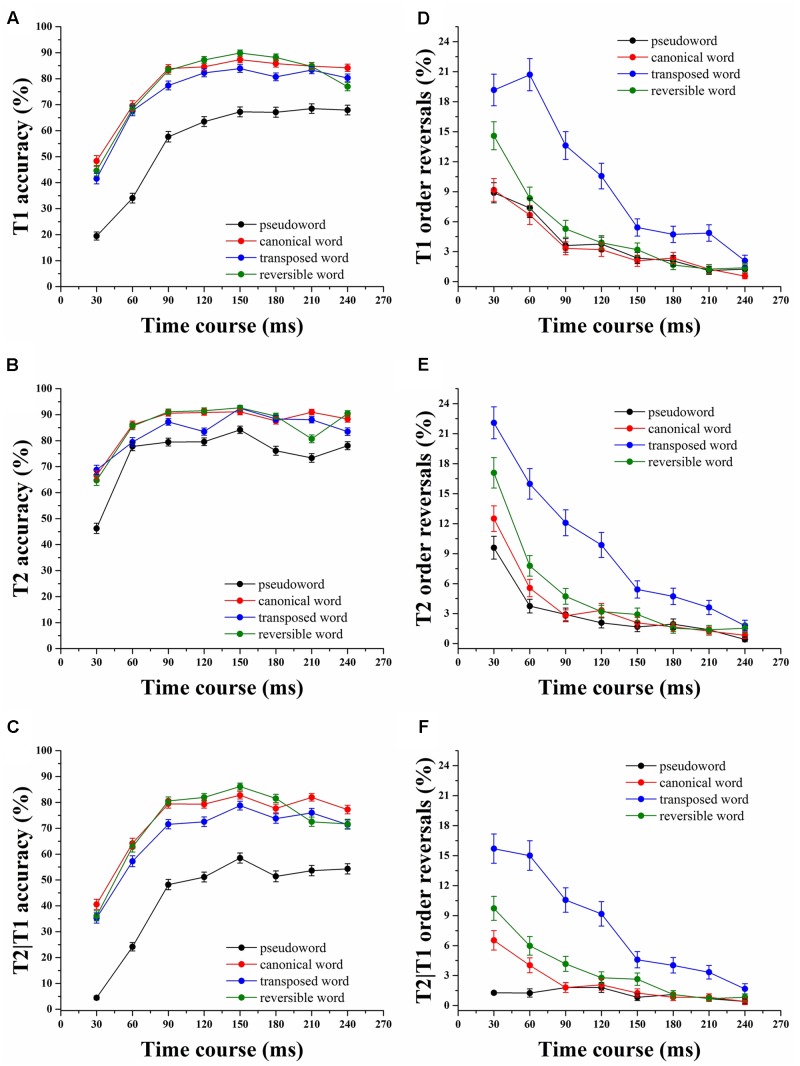
**Mean accuracy and transposition probability of T1, T2, and T2|T1 across all stimulus onset asynchrony (SOA) for the four categories.** **(A)** The mean accuracy of T1. **(B)** The mean accuracy of T2. **(C)** The mean accuracy of T2|T1. **(D)** Transposition probability of T1. **(E)** Transposition probability of T2. **(F)** Transposition probability of T2|T1. Error bars indicate the standard error of the mean.

Analyses of variance were performed by subject (*F*_1_) and item (*F*_2_) to test differences among the four stimulus conditions. The mean accuracy of identification for the first target (T1), the second target (T2) and T2 given accurate identification of T1 (T2|T1), were computed for each subject at each SOA and was averaged across participants and calculated for each stimulus category (**Figure 2**). Targets were counted as correct, regardless of the order in which they were identified. The pattern of results revealed that when T1–T2 was a compound word, regardless of the temporal order of the constituent characters, it was better identified across all SOAs than if it was a pseudoword (all *p* < 0.05), demonstrating that the T1–T2 semantic connections boost the Chinese compound words processing (**Figure 2C**). Note that the differences in T2|T1 accuracy rates between canonical words and transposed words were small but statistically significant (*F*_1(1,88)_ = 15.43, *p* < 0.001, *F*_2(7,1022)_ = 25.09, *p* < 0.001), reflecting that a transposition cost was involved in the identification of transposed words compared to canonical words during RSVP Chinese words reading (**Table 1**). From **Figures 2A,B**, T2 presented a higher performance than T1 over the SOA range of 30–240 ms in four stimulus categories (*F*_1(1,88)_ = 587.69, *p* < 0.001, *F*_2(1,1022)_ = 591.85, *p* < 0.001), and particularly in the pseudoword condition, suggesting that T2 was more likely to be identified than T1 at short SOAs.

**Table 1:** Mean accuracy in reporting T1, T2, and T2|T1 in four stimulus categories during dual-target RSVP tasks.

	Category type			
	
	Pseudo word	Canonical word	Transposed word	Reversible word
T1	54.81	78.58	74.20	77.88
T2	74.34	86.53	83.91	85.76
T2|T1	43.21	72.88	67.05	71.65


The mean accuracy in identifying T1, T2, and T2|T1 for four stimulus categories was analyzed using a 4 (category) × 8 (SOA) repeated-measures ANOVA. Identification rates across conditions with a main effect of SOA were observed for T1 (*F*_1(7,308)_ = 440.33, *p* < 0.001, *F*_2(7,3577)_ = 446.76, *p* < 0.001), T2 (*F*_1(7,308)_ = 183.75, *p* < 0.001, *F*_2(7,3577)_ = 183.18, *p* < 0.001) and T2|T1 (*F*_1(7,308)_ = 424.79, *p* < 0.001, *F*_2(7,3577)_ = 371.05, *p* < 0.001), as well as a main effect of category for T1 (*F*_1(3,132)_ = 128.13, *p* < 0.001, *F*_2(3,1533)_ = 192.34, *p* < 0.001), T2 (*F*_1(3,132)_ = 62.31, *p* < 0.001, *F*_2(3,1533)_ = 86.69, *p* < 0.001) and T2|T1 (*F*_1(3,132)_ = 176.12, *p* < 0.001, *F*_2(3,1533)_ = 11.51, *p* < 0.001). The interaction between SOA and category was significant for T1 (*F*_1(21,924)_ = 7.06, *p* < 0.001, *F*_2(21,10731)_ = 7.07, *p* < 0.001), T2 (*F*_1(21,924)_ = 6.84, *p* < 0.001, *F*_2(21,10731)_ = 6.82, *p* < 0.001) and T2|T1 (*F*_1(21,924)_ = 5.82, *p* < 0.001, *F*_2(21,10731)_ = 6.04, *p* < 0.001). A *post hoc* multiple comparisons test revealed remarkable differences between the canonical, transposed and reversible words compared with the pseudowords in four stimulus categories (all *p* < 0.001) for all T1, T2, and T2|T1 conditions. The data, taken together, suggested that the constituent characters of compound words, regardless of their order, boost identification of a whole word.

The proporation of order reversals for T1, T2, T2|T1 was highest in the transposed word condition and lowest in the pseudoword condition (**Table 2**). We are mainly concerned with the transposition probability of T2|T1 in four stimulus categories. The highest proportion of order reversals occurs at 30 ms (all *p* < 0.001), drops precipitously and approaches the inflection point until the SOA of 180 ms in canonical and reversible words (two-tailed *t*-test, paired samples, *p* > 0.05), indicating that character order errors for canonical and reversible words are mainly encoded during the time period from 30 to 180 ms (**Figure 2F**). However, it is still dropping until the SOA of 240 ms in the transposed word condition, revealing more interference between the two constituent characters. Subsequent paired-samples *t*-test indicate no significant differences among the four stimulus conditions at the SOA of 240 ms (all *p* > 0.05), implying that character order and T1–T2 relatedness is no longer a factor. ANOVAs were also carried out to reveal the proportion of order reversals for T1, T2, and T2|T1 in four categories. The main effect of the condition was significant for T1 (*F*_1(3,132)_ = 61.74, *p* < 0.001, *F*_2(3,1533)_ = 81.65, *p* < 0.001), T2 (*F*_1(3,132)_ = 56.50, *p* < 0.001, *F*_2(3,1533)_ = 76.58, *p* < 0.001) and T2|T1 (*F*_1(3,132)_ = 79.66, *p* < 0.001, *F*_2(3,1533)_ = 112.93, *p* < 0.001), as was the main effect of SOA for T1 (*F*_1(7,308)_ = 102.53, *p* < 0.001, *F*_2(7,3577)_ = 107.49, *p* < 0.001), T2 (*F*_1(7,308)_ = 131.65, *p* < 0.001, *F*_2(7,3577)_ = 138.02, *p* < 0.001) and T2|T1 (*F*_1(7,308)_ = 61.60, *p* < 0.001, *F*_2(7,3577)_ = 65.89, *p* < 0.001). More importantly, the interaction between the two factors was also significant for T1 (*F*_1(21,924)_ = 7.39, *p* < 0.001, *F*_2(21,10731)_ = 7.75, *p* < 0.001), T2 (*F*_1(21,924)_ = 6.68, *p* < 0.001, *F*_2(21,10731)_ = 7.01, *p* < 0.001) and T2|T1 (*F*_1(21,924)_ = 10.16, *p* < 0.001, *F*_2(21,10731)_ = 10.87, *p* < 0.001). *Post hoc* multiple comparison tests revealed striking differences in the order reversals of T1, T2, and T2|T1 between the transposed and reversible categories compared to the pseudoword category (all *p* < 0.05). Additionally, the subjects’ order reversal in the transposed category was significantly higher than in the canonical and reversible categories (*p* < 0.001), suggesting that severe competition occurred between the two characters, and the order information was lost.

**Table 2:** Average transposition probability of T1, T2, and T2|T1 in four stimulus categories in dual-target RSVP tasks.

	Category type			
	
	Pseudo word	Canonical word	Transposed word	Reversible word
T1	3.80	3.58	10.14	4.95
T2	2.97	3.75	9.44	5.02
T2|T1	1.01	2.22	8.00	3.49


## Discussion

The present study explores the character decomposition and transposition processes of two-character Chinese compound words and pseudowords in dual-target RSVP. Our findings indicate that the T1–T2 semantic relationship between the two constituent morphemes facilitates the identification of the Chinese compound words in three word conditions. There is a transposition cost in identifying transposed words in comparison to canonical words. The character order errors in Chinese compound words mainly occurred during the initial stage of visual word processing (30–180 ms for canonical and reversible words, 30–240 ms for transposed words).

The first question addressed here is that the character decomposition and transposition processes of compound words have an impact on visual word recognition in RSVP reading. Better T2|T1 performance for the compound words indicates they are processed as a whole, and the semantic connections between the two constituent characters boost the identification of Chinese compound words compared with pseudowords, regardless of the character order (**Figure 2C**). It is noted that the statistical results reveal that the T2|T1 accuracy rate of transposed words is significantly lower than that of canonical words over the SOAs range of 30–240 ms (**Figure 2C**), indicating a transposition cost associated with transposed characters. The character transpositions disrupt the relational structure (i.e., character order) of the base word, therefore, a character order process occurred in the processing of transposed words. Although character transpositions resulted in some cost of reading, the visual similarity of the two constituents between the transposed word and corresponding canonical words guarantees a minimum amount of correct bottom–up input for word processing. Therefore, the identification accuracy of transposed words was significantly higher than that of pseudowords (**Figure 2C**). Additionally, this facilitation between the two characters might be due to the selectivity of lexical cohorts. The first characters may limit the cohort of possible candidates for the second character to only those that have semantic connections with the activated first characters. Hence, the facilitation effect of the second characters was obtained in the compound word condition.

Taken together, these results demonstrate that both character combinations and the access to the individual constituent character meaning contribute to the identification of Chinese compound words. However, a transposition cost is involved in identifying transposed words compared to canonical words during the character decomposition and transposition processes of Chinese compound words.

The second question addressed in the present study is the time course of character order errors during two-character compound words processing. Owing to the temporal characteristics of the RSVP paradigm, observers may reverse the temporal order of the two targets (Chun and Potter, 1995; Cao H. et al., 2016). The episodic distinctiveness hypothesis proposes that sustained attention may allow for accurate reporting of the successive target characters, but observers have difficulty in reporting the correct order if memory representations between the two separately presented targets lack episodic distinctiveness (Wyble et al., 2009). In our study, when the two components of word pairs are sequentially presented in the RSVP stream (30 ms/item), participants have a strong impression of seeing them, but they sometimes cannot differentiate the actual order, particularly for the transposed words (**Figure 2F**). Additionally, the highest proportion of order reversals occurs at 30 ms and drops precipitously until approximately 180 ms for canonical and reversible words, but 240 ms for transposed words, demonstrating severe competition and combination representation between the two constituent components of compound words. Such integration improves identification of both lexical related characters, resulting in the loss of temporal order information and an increase in order errors. Importantly, the proportion of order reversals decreased as the SOA increases and converged at 240 ms in all compound word categories. Taken together, the findings demonstrated that the holistic representation of the base word was activated, however, the order of the two constituent morphemes was not strictly processed during the very early stage of visual word processing.

Our previous study about the character decomposition and transposition processes of two-character Chinese compound words and pseudowords showed that, the AB occurred when two characters could not be integrated into a single compound word (pseudoword condition), but the ABs were eliminated when two characters could be integrated into a single compound word regardless of their orders (Cao H. et al., 2016). However, the T2 performance was typically better than T1 in all conditions when the characters were fast sequentially presented at a rate of 30 ms/item, indicating that the attentional blink effect was not obtained in the current study. We deduce that the extremely rapid serial character presentation disturbed the AB pattern, and observers tended to easily identify the second character in immediate memory.

## Author Contributions

Conceived and designed the experiments: H-MY and H-WC. Performed the experiments: H-WC. Analyzed the data: H-WC. Contributed reagents/materials/analysis tools: H-WC and K-YY. Wrote the paper: H-WC, K-YY, and H-MY.

## Conflict of Interest Statement

The authors declare that the research was conducted in the absence of any commercial or financial relationships that could be construed as a potential conflict of interest.
